# Supplementary material for: Splice-Junction-Based Mapping of Alternative Isoforms in the Human Proteome
Source: Cell Rep. Author manuscript; Available in PMC 2020 Jan 15. (PMC6961840; doi:10.1016/j.celrep.2019.11.026)
Supplement: 2 [file NIHMS1546469-supplement-2.zip › DF1/PXD010154/Esophagus/Esophagus_23_CETN2_TSLGNEGPGLMASSSQR.pdf]

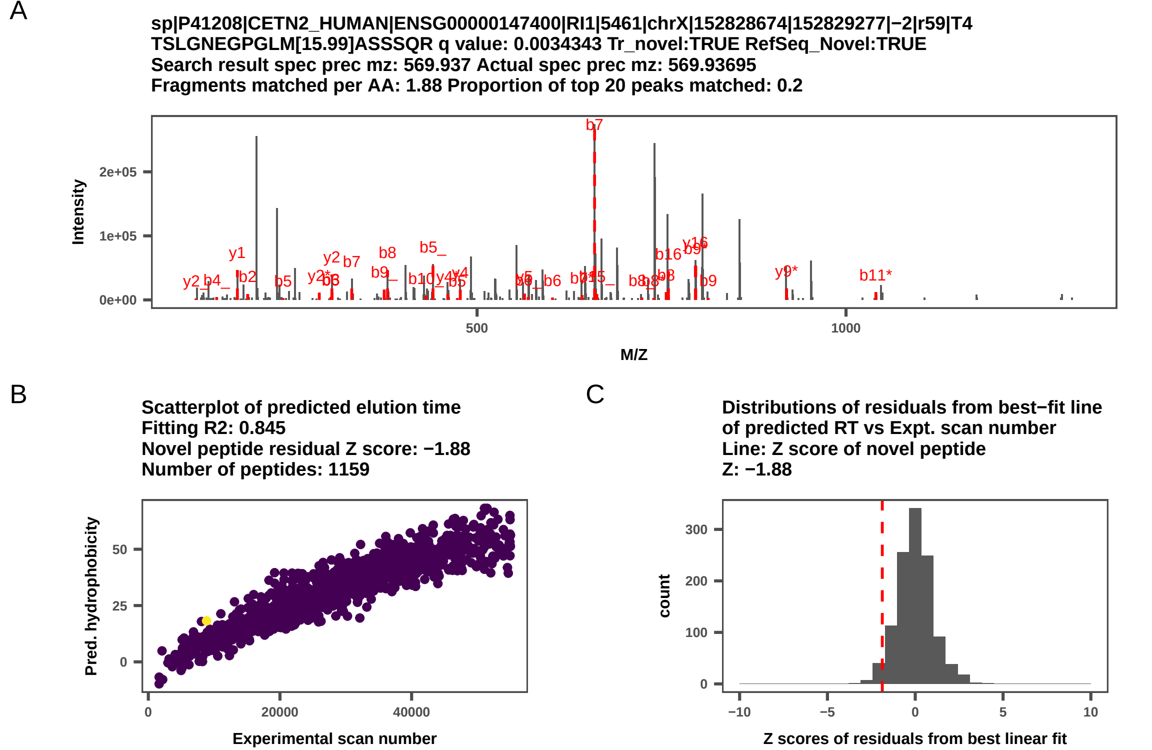
A
sp|P41208|CETN2_HUMAN|ENSG00000147400|RI1|5461|chrX|152828674|152829277|−2|r59|T4
TSLGNEGPGLM[15.99]ASSSQR q value: 0.0034343 Tr_novel:TRUE RefSeq_Novel:TRUE
Search result spec prec mz: 569.937 Actual spec prec mz: 569.93695
Fragments matched per AA: 1.88 Proportion of top 20 peaks matched: 0.2
Intensity
M/Z
B
Scatterplot of predicted elution time
Fitting R2: 0.845
Novel peptide residual Z score: −1.88
Number of peptides: 1159
Pred. hydrophobicity
Experimental scan number
C
Distributions of residuals from best−fit line
of predicted RT vs Expt. scan number
Line: Z score of novel peptide
Z: −1.88
count
Z scores of residuals from best linear fit
